# Supplementary material for: A transient protein folding response targets aggregation in the early phase of TDP-43-mediated neurodegeneration
Source: Nat Commun. 2024 Feb 19;15:1508. doi: 10.1038/s41467-024-45646-9 (PMC10876645; doi:10.1038/s41467-024-45646-9)
Supplement: Supplementary file 3 — Description of Additional Supplementary Files [file 41467_2024_45646_MOESM3_ESM.pdf]

Description of Additional Supplementary Files

File Name: Supplementary Data 1
Description: Temporal quantitative proteomics control and rNLS cortex overall results.

File Name: Supplementary Data 2
Description: Quantitative proteomics control and rNLS cortex pairwise comparisons at each timepoint.

File Name: Supplementary Data 3
Description: Common and unique proteins up and down at each timepoint from Venn diagram.

File Name: Supplementary Data 4
Description: All metascape gene ontology analysis, cortex timepoint, WCNA modules, Dnajb5 knockout cortex.

File Name: Supplementary Data 5
Description: WPCNA and PloGO gene ontology of control and rNLS cortex proteomics.
