# Supplementary material for: A transient protein folding response targets aggregation in the early phase of TDP-43-mediated neurodegeneration
Source: Nat Commun. 2024 Feb 19;15:1508. doi: 10.1038/s41467-024-45646-9 (PMC10876645; doi:10.1038/s41467-024-45646-9)
Supplement: Supplementary file 9 — Reporting Summary [file 41467_2024_45646_MOESM9_ESM.pdf]

nature portfolio

Corresponding author(s): Adam Walker

Last updated by author(s): Jan 25, 2024

Reporting Summary

Nature Portfolio wishes to improve the reproducibility of the work that we publish. This form provides structure for consistency and transparency in reporting. For further information on Nature Portfolio policies, see our Editorial Policies and the Editorial Policy Checklist.

Statistics

For all statistical analyses, confirm that the following items are present in the figure legend, table legend, main text, or Methods section.

n/a	Confirmed	
☐	☒	The exact sample size (*n*) for each experimental group/condition, given as a discrete number and unit of measurement
☐	☒	A statement on whether measurements were taken from distinct samples or whether the same sample was measured repeatedly
☐	☒	The statistical test(s) used AND whether they are one- or two-sided<br>*Only common tests should be described solely by name; describe more complex techniques in the Methods section.*
☒	☐	A description of all covariates tested
☐	☒	A description of any assumptions or corrections, such as tests of normality and adjustment for multiple comparisons
☐	☒	A full description of the statistical parameters including central tendency (e.g. means) or other basic estimates (e.g. regression coefficient) AND variation (e.g. standard deviation) or associated estimates of uncertainty (e.g. confidence intervals)
☐	☒	For null hypothesis testing, the test statistic (e.g. *F*, *t*, *r*) with confidence intervals, effect sizes, degrees of freedom and *P* value noted<br>*Give P values as exact values whenever suitable.*
☒	☐	For Bayesian analysis, information on the choice of priors and Markov chain Monte Carlo settings
☐	☒	For hierarchical and complex designs, identification of the appropriate level for tests and full reporting of outcomes
☒	☐	Estimates of effect sizes (e.g. Cohen's *d*, Pearson's *r*), indicating how they were calculated

Our web collection on statistics for biologists contains articles on many of the points above.

Software and code

Policy information about availability of computer code

Data collection	Light Cycler 480 Software Release 1.5.1.62 SP3 Roche RRID:SCR_012155<br>Image Studio Lite version 5.2.5 Li-Cor RRID:SCR_013715<br>Zen software Zeiss RRID:SCR_013672<br>Activity Monitor 7 Software Med Associates RRID:SCR_014296
Data analysis	CellProfiler version 4.2.1 Broad Institute RRID:SCR_007358<br>Prism version 10 GraphPad Software RRID:SCR_002798<br>Metascape Zhou, Y., et al. Metascape provides a biologist-oriented resource for the analysis of systems-level datasets. Nat Commun 10, 1523 (2019) RRID:SCR_016620<br>Ingenuity Pathway Analysis Qiagen RRID:SCR_008653<br>Proteome Discoverer V2.1 ThermoFisher Scientific RRID:SCR_014477<br>FIJI software (v1.53c) National Institutes of Health RRID:SCR_002285<br>Adobe Illustrator (v25.3.1) Adobe Systems Incorporated RRID:SCR_010279<br>R Studio RRID:SCR_000432<br>TMTPrepPro package Mirzaei, M., et al. TMT one-stop shop: from reliable sample preparation to computational analysis platform. Methods Mol Biol 1549, 45-66 (2017)<br>Weighted gene correlation network analysis package RRID:SCR_003302<br>Shiny package RRID:SCR_001626<br>Tidyverse RRID:SCR_019186

ggrepel RRID:SCR_017393
readxl RRID:SCR_018083
ComplexHeatmap package RRID:SCR_017270

For manuscripts utilizing custom algorithms or software that are central to the research but not yet described in published literature, software must be made available to editors and reviewers. We strongly encourage code deposition in a community repository (e.g. GitHub). See the Nature Portfolio guidelines for submitting code & software for further information.

Data

Policy information about availability of data

All manuscripts must include a data availability statement. This statement should provide the following information, where applicable:

- Accession codes, unique identifiers, or web links for publicly available datasets
- A description of any restrictions on data availability
- For clinical datasets or third party data, please ensure that the statement adheres to our policy

The mass spectrometry proteomics data have been deposited to the ProteomeXchange Consortium via the PRIDE115 partner repository with the dataset identifier PXD042382.

Source data for all figures and supplementary figures, and supplementary tables are provided with this paper and any additional information required to re-analyze the data reported in this paper is available from the lead contact upon request.

Research involving human participants, their data, or biological material

Policy information about studies with human participants or human data. See also policy information about sex, gender (identity/presentation), and sexual orientation and race, ethnicity and racism.

Reporting on sex and gender	Human postmortem tissue was analysed from n = 3 non-neurologically diseased and n = 3 ALS or ALS and FTD cases. The sex is reported for each case in Table 2.
Reporting on race, ethnicity, or other socially relevant groupings	N/A
Population characteristics	Table 2 also provides information the clinical diagnosis of each case, sex, age at onset, age at death, site of onset, brain weight (g) and post-mortem delay (h)
Recruitment	This study used post-mortem brain tissue that was collected by Maurice A. Curtis, Richard L. M. Faull and banked by the Neurological Foundation of New Zealand Human Brain Bank. The donors donated their brain voluntarily and there was no bias in the recruitment of samples to the study.
Ethics oversight	Experiments were conducted with informed donor consent, with approval from the NZ Health and Disability Ethics Committee (14/NTA/208) and The University of Auckland Human Participants Ethics Committee.

Note that full information on the approval of the study protocol must also be provided in the manuscript.

Field-specific reporting

Please select the one below that is the best fit for your research. If you are not sure, read the appropriate sections before making your selection.

☒ Life sciences ☐ Behavioural & social sciences ☐ Ecological, evolutionary & environmental sciences

For a reference copy of the document with all sections, see nature.com/documents/nr-reporting-summary-flat.pdf

Life sciences study design

All studies must disclose on these points even when the disclosure is negative.

Sample size	GPower (version 3.1.9.6) was used. To determine the difference between two means, we used a two-tailed test, a = 0.05, and power of 80%. Our calculations indicate that a group size of 13 mice is sufficient.
Data exclusions	If mice were lost from a study for reasons unrelated to the development of disease, the data from these mice were removed from all analyses.
Replication	Extensive measures were taken to ensure the reproducibility of results. For proteomics of the rNLS8 cortex n = 5 mice per group was analysed. For proteomics of DNAJB5KO/KO mouse cortex n = 3 mice per group was analysed. For animal behaviour n = 9-21 mice per group was analysed. For experiments in cell and primary neuronal culture n = 3-5 independent experiments were conducted to ensure consistency. Lastly, for DNAJB5 proof-of-concept experiments three model system were employed, HEK293, primary cortical neurons, and in vivo (mice). All attempts at replicating results of each experiment were successful.
Randomization	For AAV mouse studies, viral injections were performed prior to genotyping of animals and genotypes were determined independently from experimenters performing animal assessment and behavioural testing, therefore pups were assigned randomly to each of the experimental

groups. Covariates such as age were accounted for by injecting all mice with AAV at postnatal day 0.

Blinding

Investigators were blinded to treatment AAV group and genotype of mice in experiments related to DNAJB5 KO. Experimentors were blinded throughout behavioural test data collection and analysis of data was conducted with blinded groups only to be unblinded at the completion of the experiments.

Reporting for specific materials, systems and methods

We require information from authors about some types of materials, experimental systems and methods used in many studies. Here, indicate whether each material, system or method listed is relevant to your study. If you are not sure if a list item applies to your research, read the appropriate section before selecting a response.

Materials & experimental systems

n/a	Involved in the study	
☐	☒	Antibodies
☐	☒	Eukaryotic cell lines
☒	☐	Palaeontology and archaeology
☐	☒	Animals and other organisms
☒	☐	Clinical data
☒	☐	Dual use research of concern
☒	☐	Plants

Methods

n/a	Involved in the study	
☒	☐	ChIP-seq
☒	☐	Flow cytometry
☒	☐	MRI-based neuroimaging

Antibodies

Antibodies used

TDP-43, pAb, 600 ug/mL Proteintech Cat# 10782-2-AP, RRID:AB_615042
Immunoblot = 0.12 ug/mL

phosphoTDP-43 (S403/S404), pAb, no conc, Cosmo Bio Cat# CAC-TIP-PTD-P05, RRID:AB_1961902
Immunoblot = 1:1000 dil.

phosphoTDP-43 (S409/S410), mAb, 1.0 mg/mL, BioLegend Cat# 829901, RRID:AB_2564934
Immunoblot = 1 ug/mL
Immunohistochemistry (human) = 0.33 mg/mL

FLAG, mAb, 1 mg/mL, Sigma-Aldrich Cat# F1804, RRID:AB_262044
Immunoblot = 1 ug/mL
Immunocytochemistry = 1 ug/mL

FLAG, mAb, 0.5 mg/mL Genscript Cat# A00187S RRID:AB_1720813
Immunoprecipitation = 1 ug/mL

TDP-43, mAb, 1.55mg/ml, #5117 CNDR
Immunohistochemistry = 0.5 ug/mL

NeuN, pAb, 1 mg/mL, Millipore Cat# ABN91, RRID:AB_11205760
Immunohistochemistry = 2 ug/mL

DNAJB6, pAb, 180 ug/mL, Proteintech Cat# 11707-1-AP, RRID:AB_2230757
Immunoblot = 0.18 ug/mL
Immunohistochemistry = 0.18 ug/mL

HSPH1, pAb, 1 mg/mL, Aviva Systems Biology Cat# OAAF01833, RRID:AB_2631006
Immunoblot = 1 ug/mL
Immunohistochemistry = 10 ug/mL

DCTN2, pAb, 300 ug/mL, Proteintech Cat# 11361-1-AP, RRID:AB_2090609
Immunoblot = 0.3 ug/mL
Immunohistochemistry = 3 ug/mL

PARP1, pAb, 300 ug/mL, GeneTex Cat# GTX100573, RRID:AB_1241155
Immunoblot = 0.3 ug/mL
Immunohistochemistry = 0.6 ug/mL

aB-crystallin (HSPB5) pAb, 350 ug/mL, Proteintech Cat# 15808-1-AP, RRID:AB_2292175
Immunoblot = 0.35 ug/mL * Ab did not work

Immunohistochemistry = 0.7 ug/mL

IFIT3, pAb, 600 ug/mL, Proteintech Cat# 15201-1-AP, RRID:AB_2248738
Immunohistochemistry = 6 ug/mL *Ab did not work

DNAJB5, pAb, 800 ug/mL, Proteintech Cat# 16453-1-AP, RRID:AB_2094724
Immunohistochemistry (mouse) = 1.6 mg/mL
Immunohistochemistry (human) = 8 mg/mL

HSP90AB1, pAb, 1 mg/mL, Abcam Cat# ab2927, RRID:AB_303422
Immunoblot = 1 ug/mL
Immunohistochemistry = 10 ug/mL

CDC37, mAb, no conc, Cell Signaling Technology Cat# 4793, RRID:AB_10695539
Immunoblot = 1:1000
Immunohistochemistry = 1:100

CALR, pAb, 200 ug/mL, GeneTex Cat# GTX111627, RRID:AB_11175861
Immunoblot = 0.4 ug/mL *Ab did not work
Immunohistochemistry = 2 ug/mL

HSPA1A, 1 mg/mL Enzo ADA-SPA-810
Immunoblot = 1 mg/mL

GFP, pAb, 5 mg/mL Abcam Cat# ab290 RRID:AB_303395
Immunoprecipitaton = 5 ug/mL

GFAP, pAb, 2.9 mg/mL Agilent Cat# z0334 RRID:AB_10013382
Immunoblot = 0.29 mg/mL
Immunohistochemistry = 1.45 mg/mL

Secondary antibodies
Donkey anti-mouse AlexaFluor 647 secondary, Thermo Fisher Scientific Cat# A-31571, RRID:AB_162542
Immunocytochemistry = 2 ug/mL

Goat anti-rabbit AlexaFluor 555 secondary, Thermo Fisher Scientific Cat# A-21429, RRID:AB_2535850
Immunohistochemistry = 2 ug/mL

Goat anti-chicken AlexaFluor 488 secondary, Abcam Cat# ab150169, RRID:AB_2636803
Immunohistochemistry = 2 ug/mL

Goat anti-mouse IgG 680 secondary, LI-COR Biosciences Cat# 926-68070, RRID:AB_10956588
Immunoblot = 50 ng/mL

Goat anti-rabbit IgG 800 secondary, LI-COR Biosciences Cat# 926-32211, RRID:AB_621843
Immunoblot = 50 ng/mL

Goat anti-rat IgG 800 secondary, LI-COR Biosciences Cat# 926-32219, RRID:AB_1850025
Immunoblot = 50 ng/mL

Goat anti-rabbit IgG AlexaFluor® 488 Jackson ImmunoResearch Cat# 111-545-144 RRID:AB_2338052
Immunohistochemistry = 4 mg/mL

Goat anti-rabbit IgG AlexaFluor® 647 Jackson ImmunoResearch Cat# 112-605-167
Immunohistochemistry = 4 mg/mL RRID:AB_2338404

Validation	All commercial primary and secondary antibodies were validated according to respective manufacturer's information and citations. All antibodies were used at concentrations recommended by the specification sheet and the concentrations used for immunohistochemistry, immunoblotting, and immunocytochemistry are listed above for each antibody.<br><br>The only antibody that is not currently commercially available is TDP-43, mAb, 1.55mg/ml, #5117 CNDR, and this was validated in the following paper: Kwong, L.K., et al. Novel monoclonal antibodies to normal and pathologically altered human TDP-43 proteins. Acta Neuropathol Commun 2, 33 (2014)<br><br>Where antibodies did not produce a signal, we tested three increasing concentrations (directed by the specification sheet), for example, for anti-calreticulin GeneTex Cat# GTX111627, RRID:AB_11175861, we tested by immunoblot at 0.4 ug/mL, 0.2 ug/mL, and 0.067 ug/mL but none of these produced a signal on the immunoblot. For the same antibody, we optimised the immunohistochemistry concentrations at 2 ug/mL, 0.2 ug/mL, and 0.1 ug/mL and found that 2 ug/mL gave specific staining. Validation of DNAJB5 antibody was performed using wildtype, hemi- and homozygous Dnajb5 knockout mouse tissue, shown in Supplementary Figure 6.

Eukaryotic cell lines

Policy information about cell lines and Sex and Gender in Research

Cell line source(s)	HEK293T1/17 cells (female origin) ATCC Cat #: CRL-11268 RRID:CVCL_1926<br>Primary cortical neuronal culture from wild type C57BL/6 × SJL F1 mice (embryonic day 15)
Authentication	HEK293T1/17 cells were obtained from the ATCC and are observed to maintain expected morphology but have not been additionally genetically authenticated since purchase in 2018. All experiments were conducted from cells thawed from an expanded population of cells that were initially frozen after purchase from ATCC.
Mycoplasma contamination	Cells in culture were negative for mycoplasma with routine testing. MycoAlert mycoplasma detection kit from Lonza LT07-218.
Commonly misidentified lines<br>(See ICLAC register)	No commonly misidentified lines were used in this research.

Animals and other research organisms

Policy information about studies involving animals; ARRIVE guidelines recommended for reporting animal research, and Sex and Gender in Research

Laboratory animals	Timed mates: C57BL/6 × SJL F1 mice (Embryonic day 15 for neuronal cultures) RRID:MGI:5655052<br>tetO-hTDP-43-ΔNLS line 4 Jackson Laboratory RRID:IMSR_JAX:014650 (Animals were aged to 8 weeks on Dox and then tissue was collected at the following timepoints off Dox; 1, 2, 4, 6 weeks off Dox and recovery mice were 6 weeks off Dox + 2 weeks back on Dox)<br>NEFH-tTA line 8 Jackson Laboratory RRID:IMSR_JAX:025397 (Animals were aged to 8 weeks on Dox and then tissue was collected at the following timepoints off Dox; 1, 2, 4, 6 weeks off Dox and recovery mice were 6 weeks off Dox + 2 weeks back on Dox)<br>C57BL/6NJ-Dnajb5em1(IMPC)J/Mmjax Jackson Laboratory RRID:MMRRC_043738-JAX (3 months)<br>TDP43Q331K mice Jackson Laboratory RRID:IMSR_JAX:017933 (3 months and 16 months)
Wild animals	No wild animals were used in this research.
Reporting on sex	Female and male mice were used in these studies and sex of each animal used in proteomics studies is detailed in Supplementary Table 1. Data was only disaggregated for sex where appropriate, for example Supplementary Figure 14, where weights of males and females were analyzed separately.
Field-collected samples	No filed collected samples were used in this research.
Ethics oversight	All experiments were carried out in accordance with the Australian Code of Practice for the Care and Use of Animals for Scientific Purposes (8th Edition, 2013). Mice were both bred and housed for all subsequent in vivo mouse studies in a Specified Pathogen-Free (SPF) animal facility with a 12 h light/dark cycle (lights on at 06:00 h) and the room temperature and humidity maintained at 21 ± 1°C and 55 ± 5%, respectively. Experiments were conducted with approval from the Animal Ethics Committees of Macquarie University (#2016-026) and The University of Queensland (QBI/131/18 and 2021-AE000451).

Note that full information on the approval of the study protocol must also be provided in the manuscript.
